# Interferon-γ and Hypoxia Priming Have Limited Effect on the miRNA Landscape of Human Mesenchymal Stromal Cells-Derived Extracellular Vesicles

**DOI:** 10.3389/fcell.2020.581436

**Published:** 2020-12-15

**Authors:** Juliette Peltzer, Kyle Lund, Marie-Emmanuelle Goriot, Marion Grosbot, Jean-Jacques Lataillade, Philippe Mauduit, Sébastien Banzet

**Affiliations:** ^1^Institut de Recherche Biomédicale des Armées, Clamart, France; ^2^UMR-MD-1197, INSERM, Université Paris 11, Ministère de la défense, Villejuif, France

**Keywords:** mesenchymal stromal cells, extracellular vesicles, miRNA, priming, exosome (vesicle), mesenchymal stem cells

## Abstract

Mesenchymal stromal cell (MSC)-based cell therapy has received great interest in regenerative medicine. Priming the cells during the culture phase can improve their efficacy and/or survival after injection. The literature suggests that MSC extracellular vesicles (EV) can recapitulate a substantial part of the beneficial effects of the cells they originate from, and that micro-RNAs (miRNAs) are important players in EV biological action. Here, our aim was to determine if two classical priming methods of MSC, interferon-gamma (IFNγ) and hypoxia (HYP), could modify their EV miRNA content. Human bone marrow MSCs (BM-MSCs) from five healthy donors were cultured with IFNγ or in HYP or in control (CONT) conditions. The conditioned media were collected after 48 h in serum-free condition and EV were isolated by ultracentrifugation. Total RNA was isolated, pools of CONT, IFN, and HYP cDNA were prepared, and a miRNA profiling was performed using RT-qPCR. Then, miRNAs were selected based on their detectability and measured on each individual EV sample. Priming had no effect on EV amount or size distribution. A set of 81 miRNAs was detected in at least one of the pools of EVs. They were measured on each individual sample; 41 miRNAs were detected in all samples. The principal component analysis (PCA) failed to discriminate the groups. HYP induced a significant decrease in EV hsa-miR-34a-3p content and IFN induced a significant increase in five miRNAs (hsa-miR-25-3p, hsa-miR-106a-5p, hsa-miR-126-3p, hsa-miR-451a, and hsa-miR-665). Taken together, we found only limited alterations in the miRNA landscape of MSC EV with a high inter-individual variability.

## Introduction

Mesenchymal stromal cells (MSCs) are adult, non-hematopoietic cells first identified in the bone marrow. They are found in numerous other tissues such as adipose tissue, muscle, gingiva, as well as in perinatal tissues (umbilical cord or placental membranes). MSCs have been studied for their clinical potential, specifically in the field of inflammation and tissue repair (for review, see Dimarino et al., 2013; Galipeau and Sensébé, 2018). Although they were first thought to differentiate in other cell types, it is now widely accepted that, except for bone, most of their beneficial effects are due to their paracrine function. Many studies have demonstrated their immunomodulatory, anti-apoptotic, pro-angiogenic, or anti-fibrotic effects, widely mediated by secreted bioactive molecules (da Silva Meirelles et al., 2009; de Almeida et al., 2013). MSC’s secretome is composed of soluble factors and extracellular vesicles (EV) that both contribute to their biologic effects (Spees et al., 2016; Mitchell et al., 2019). MSC-derived EV are nano-sized (50–1000 nm) vesicles delimited by a bi-lipid layer, that include different types of vesicles such as exosomes or ectosomes. EV contain proteins, mRNAs, micro-RNAs (miRNAs) that reproduce a substantial part of the biological effects of the cells they originate from. Therefore, EV have been proposed as a surrogate for MSC therapy, easier to produce and store, and immediately available.

Micro-RNAs are small non-coding RNAs involved in the post-transcriptional control of gene expression by targeting specific mRNAs, blocking their translation, or activating their degradation. Some miRNAs are actively secreted in EV and they contribute to the therapeutic effects of MSC-derived EV (Mead and Tomarev, 2017; Fujii et al., 2018; Xiao et al., 2018). The MSC-EV miRNA landscape has been described and the analysis of the 23 top expressed miRNA shows their involvement in pathways that are crucial for tissue repair (angiogenesis, integrin signaling, Wtn signaling, or TGF-β signaling) (Ferguson et al., 2018). Therefore, miRNAs are considered potential players of the MSC-EV, even if this view is still debated because of the low amount of miRNA reported in some studies (Toh et al., 2018).

MSCs are able to modulate their environment, as observed in the niches they come from, but they are also responsive to their environment. MSC function can be improved by modulating the cell culture conditions, a process known as preconditioning or priming. Priming could both prepare the cells to the environment they will be injected in (thereby improving their survival) and their biological activity for a specific pathology (Shojaei et al., 2019). Therefore, hypoxia (HYP) priming has been successfully used for MSC treatment in ischemic diseases (Leroux et al., 2010; Jaussaud et al., 2013; Zhang et al., 2019), improving both survival and function (Chacko et al., 2010; Peterson et al., 2011; Beegle et al., 2015; Feng and Wang, 2017; Elabd et al., 2018). Similarly, inflammatory priming with TNFα, IL1β, or interferon gamma (IFNγ) priming increase the immunomodulatory properties of MSC *in vitro* and *in vivo* (English et al., 2007; Chinnadurai et al., 2014; Cassano et al., 2018; Baudry et al., 2019; Magne et al., 2020). Thus, MSC priming is currently considered an important way to improve MSC function or get rid of inter-individual variability.

A substantial part of MSC function relies on their paracrine secretion and it has been shown that priming the cells during the culture modifies their secretome. Culturing MSC under HYP led to changes in the proteins they secrete (Paquet et al., 2015; Song et al., 2016). Similarly, inflammatory priming induced changes in the protein content of their secretome (Magne et al., 2020) and EVs (Zhang et al., 2018).

Despite the fact that miRNAS are important functional molecules in the EV, the effects of priming on the miRNA landscape of MSC EV have never been reported. Here, we studied miRNA in EV isolated from human bone marrow MSC (BM-MSC) cultured in standard conditions or exposed to two classical priming conditions, HYP or IFNγ.

## Methods

### MSC Culture and Priming

Human BM-MSCs were obtained after written informed consent from patients undergoing total hip replacement surgery. In accordance with the French law, prior approval by an institutional review board was not required. As previously reported (Peltzer et al., 2015), spongious bone fragments were mixed in a solution of phosphate buffered saline (PBS; PAN-Dominique Dutscher) + 1 mM EDTA (Prolabo-VWR) + ACD-A (0.32 g/l of citric acid, 0.88 g/l of sodium citrate, 0.98g/l of dextrose) + 0.5% of human serum albumin (HSA; LFB). After 20 min of settling, the supernatant was collected, centrifuged at 480 *g* for 10 min and filtered at 70 μm. The bone marrow mononuclear cells (BM-MNCs) were counted using an automated cell analyzer (Sysmex). MSCs were seeded at 4000 cell/cm^2^ in MEMα + 10% fetal calf serum (FCS, Hyclone) + 0.01 mg/ml ciprofloxacin (Bayer Pharma) and cultivated until they reached 60% of confluence. MSCs were frozen in MEMα + 10% human albumin (LFB) + 10% DMSO (Sigma). For each experiment, MSCs were thawed at passage 2 and amplified again before priming achievement. The first priming condition was HYP: MSCs were seeded at 4000 cell/cm^2^ in growth culture medium at T°= 37°C, O_2_ = 3%, humidity = 95% until they reach 70–80% of confluence. The second priming by IFNγ was carried out under the following condition: MSCs were seeded at 4000 cell/cm^2^ in growth culture medium at T°= 37°C, O_2_ = 20%, humidity = 95% until they reach 70–80% of confluence, then the cells were washed with PBS, placed in MEMα without serum, and supplemented with 25 ng/ml of IFNγ for 48 h.

To validate the efficacy of our hypoxic priming, we measured vascular endothelial growth factor (VEGF) protein by ELISA in MSC supernatant and found an increase in response to HYP (15,144 ± 3119 pg.ml^–1^ for control, 59,589 ± 42,886 pg.ml^–1^ for HYP) (data not shown). In order to validate the effectiveness of our IFN priming, we measured three cell surface markers known to be expressed at low level by MSC and stimulated by IFN. The cytometry performed on a pool of our cells showed a substantial increase in the percentage of cells expressing HLA-DR (0.59 and 88.56% for Cont and IFN, respectively), CD54 (13.56 and 99.47%), and B7-H1 (1.91 and 98.55%) (data not shown).

### EV Production and Isolation

When the cell reached confluence or at the end of priming, the media was removed and washed with PBS. Then, 30 ml of media (for 300 cm^2^ culture flask) without FCS was added for 24 h. The media was then removed of each culture flask, pooled, and the cells were counted. Different centrifugation steps were carried out: 662 *g* for 5 min followed by 1841 *g* for 5 min to remove cell debris, and then the media was centrifuged at 100,000 *g* at +4°C for 70 min. Finally, the media was removed and the EVs were resuspended at 10 μl/10^6^ MSCs.

### EV Characterization

*Transmission electron microscopy* (TEM) was performed on MSC-EV with a CM-10 microscope (Philips). The samples were placed on a carbon grid, dried at 30°C during 20 min and 2% uranyl acetate solution was added, rinsed, and the grid was dried again before imaging.

*Mass spectrometry* was used to control the presence of EV associated proteins. The MSC-EV proteins solubilized in laemmli sample buffer were separated by 10% SDS-PAGE under reducing conditions. Proteins were directly reduced, alkylated, and trypsin-digested in gel pieces. Resulting eluted peptides were analyzed by liquid chromatography mass spectrometry using nano-high pressure liquid chromatography (Ultimate 3000, Dionex) coupled with LTQ-Orbitrap Velos spectrometer (ThermoScientific, Bremen, Germany). Proteins were identified and quantified using the Proteome discoverer v1.4 software from ThermoScientific, by comparing the results with a human protein database and a control database containing scrambled sequences. Proteins identified by at least two unique peptides were further analyzed for functional enrichment using the Funrich analysis software tool^1^ and the human Funrich database.

*Nano tracking analysis* (NTA) was used to determine the EV size distribution and concentration using a Nanosight NS300 with a 488 nm laser. Samples were diluted to reach an appropriate concentration (1 × 10^8^ and 1 × 10^9^ particles.ml^–1^) with PBS. The samples were injected with an automatic syringe pump. Data acquisition and analysis were performed using the NTA Analytical Software (version 3.4). For each sample, five movies of 60 s were recorded. All others settings were in automatic mode. Particle concentration (EV.ml^–1^) was calculated following subtraction of PBS and tacking into account the sample dilution.

### Control of MSC Priming

About 1.10^5^ MSCs were incubated in PBS + 2% HSA, and 0.5% polyvalent human immunoglobulins (LFB) for 20 min at 4°C; then incubated with antibodies at a saturating concentration for 20 min at 4°C: MSC CD90, HLA-ABC, HLA-DR, B7-H1, and CD54 (all from Beckman Coulter). Then, MSCs were washed with PBS and immediately acquired using Navios cytometer (Beckman Coulter). Data were analyzed on Kaluza software (Beckman Coulter). Concentration of VEGF was determined using a sandwich-enzyme linked immunosorbent assay technique (R&D system) according to the manufacturer’s recommendations. VEGF was determined in MSC supernatant of naive and HYP-primed cells.

### EVs Phenotypic Analysis

Flow cytometry was used to analyze EV and MSC-associated markers on MSC-EV. Five μl of EVs (EV solution at 10 μl/10^6^ secreting cells) from five MSC donors were incubated with 1 μl of Aldehyde/Sulfate Latex Beads, 4% w/v, 4 μm (Molecular Probes) at room temperature for 20 min. Beads were washed with PBS + 4% of HSA (LFB) and centrifuged at 818 *g* for 5 min. EVs complexed with beads were incubated in PBS + 4% HSA for 20 min at 4°C; then incubated with antibodies at a saturating concentration for 20 min at 4°C: tetraspanin markers CD9, CD63, CD81 (Biolegend); MSC markers CD73 (BD), CD90, CD105, and hematopoietic marker CD45 (all from Beckman Coulter). Then EVs complexed beads labeled with antibodies were washed with PBS + 4% HSA and immediately acquired using Navios cytometer (Beckman Coulter). Data were analyzed on Kaluza software (Beckman Coulter).

### miRNA Measurement

Total RNA was isolated with Qiazol Lysis reagent (Qiagen) with an optimized protocol: 200 μl Qiazol and 400 μl chloroform were added to each sample and then the manufacturer’s protocol was performed with an additional precipitation step. The aqueous phase was added with an equal volume of isopropanol and 1 μl of GlycoBlue (Ambion). Total RNAs were precipitated for 20 min at 20°C. After centrifugation (12,000 *g*, 15 min, at 4°C), the pellet was washed with 70% ethanol, dried, and resuspended in 10 μl of sterile water.

cDNA was synthesized from 0.8 μl RNA in a 10 μl reaction volume with the Universal cDNA Synthesis Kit (Exiqon).

Real-time quantitative PCR (qPCR) experiments were performed with a LightCycler 480 instrument (Roche Applied Science). For miRNA profiling, pools of cDNA were made for each experimental group, 752 miRNAs were profiled in each pool with miRCURY LNA^TM^ Universal RT microRNA PCR, microRNA Ready-to-Use PCR, Human panel I and II (Exiqon). The results are not shown here but are available in a public database (GEO database, # GSE156919).

Then, we measured a selection of miRNA in each individual sample of the three groups. These miRNAs were selected based on their detectability and or their description in MSC-EV in the literature. They were measured on each individual sample using custom-designed miRCURY LNA^TM^ Universal RT microRNA PCR, Pick-&-Mix, Ready-to-use Panel (Exiqon).

All miRNAs were analyzed using geNorm version 3.4 (Vandesompele et al., 2002) to identify suitable internal references. The normalization was performed with five miRNAs, namely, hsa-let-7a-5p, hsa-miR-21-5p, 23a-3p, 23b-3p, and 24-3p. The final quantification was performed as the geometric mean of the quantification with the selected reference miRNA.

### Bioinformatic Analysis

The principal component analysis (PCA) of miRNA expression and the heatmap were performed with ClustVis (Metsalu and Vilo, 2015). The gene targets of selected miRNAs were determined using Mirtarbase 8.0 (Hsu et al., 2011). Then an enrichment analysis was performed using Panther classification system 14.0 (Mi and Thomas, 2009).

### Statistical Analysis

For individual miRNA measurement, a non-parametric test (Friedman ANOVA) was used to determine a main effect, when relevant Dunn’s multiple comparison tests were applied to compare the groups.

## Results

### EV Characterization

**FIGURE 1:**
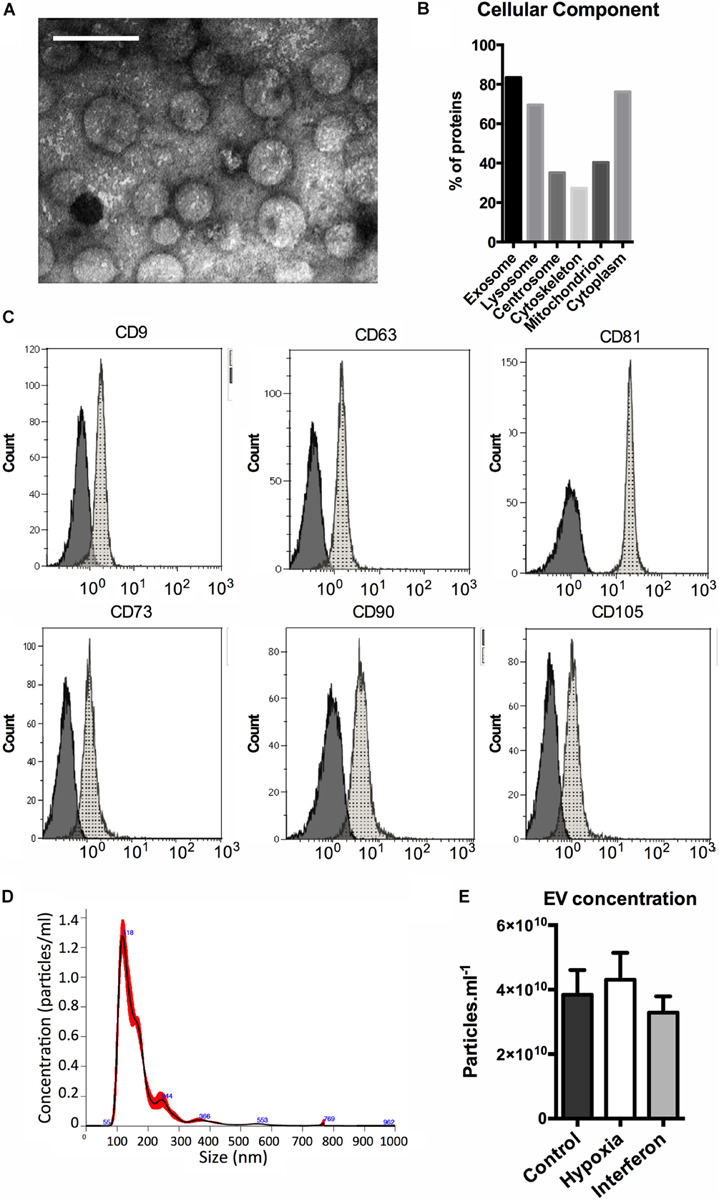
MSC extracellular vesicles characterization. **(A)** Representative transmission electron microscopy image of the MSC-EV preparation (205,000X, white scale bar = 100 nm). **(B)** Distribution of the proteins identified in control MSC-EV proteomic (LC MS-MS) analysis among cellular components (%). **(C)** Expression of EV (upper panel) and MSC (lower panel) antigens on MSC EV analyzed by flow cytometry. Representative data of median fluorescence intensities (MFIs) are shown, black histograms correspond to IgG isotype control, and gray histograms to specific antibodies antigens. **(D)** Representative nano tracking analysis (NTA) of the MSC-EV preparation showing particle concentration (*Y* axis) and size (*X* axis). EV concentration measured by NTA in the MSC-EV preparations. **(E)** Extracellular vesicles concentration in Control, Hypoxia, and Interferon-treated MSC preparations as measures by NTA (particles.ml^–1^).

The TEM analysis showed typical round-shaped pictures in EV preparations (Figure 1A). To validate our EV isolation protocol, we performed a proteomic analysis of the control MSC-EV preparation. The mass spectrometry analysis identified 185 proteins with at least two unique peptides. The results were analyzed with Funrich database (Pathan et al., 2015) and we found that 83.4% of them were annotated as “exososomal” (Figure 1B) including transmembrane (for example, HLA-A, ITA3, or ITB1) and cytosolic proteins (such as FLOT1, CAV1, ANXA 1, 2, 4, and 5).

Moreover, cytofluorimetric analyses of EV showed the presence of several antigens typically expressed by MSCs, CD73, CD90, and CD105, and of classical “exosomal” markers, the tetraspanins CD9, CD63, and CD81 (Figure 1C). The hematopoietic marker CD45 was not expressed (data not shown).

### Validation of the Priming Conditions

To validate the efficacy of our hypoxic priming, we measured VEGF protein by ELISA in MSC supernatant and found an increase in response to HYP (15,144 ± 3119 pg.ml^–1^ for control, 59,589 ± 42,886 pg.ml^–1^ for HYP) (data not shown). In order to validate the effectiveness of our IFN priming, we measured three cell surface markers expressed at low level by MSC and stimulated by IFN. The cytometry performed on a pool of our cells showed a substantial increase in the percentage of cells expressing HLA-DR (0.59 and 88.56% for Cont and IFN, respectively), CD54 (13.56 and 99.47%), and B7-H1 (1.91 and 98.55%) (data not shown).

### EV Secretion Profile

The NTA method was used to study EV concentration and size. A typical size/concentration profile is shown (Figure 1D). The EV concentrations in the final preparation were slightly different. We found a significant global effect when comparing the three groups; however, no difference between control and HYP or control and IFN priming was found in the multiple comparison test (Figure 1E). The NTA showed that priming did not alter EV size distribution as evaluated by the mode, D10, D50, and D90 (data not shown).

### miRNA Profiling in Pools of EV

**FIGURE 2:**
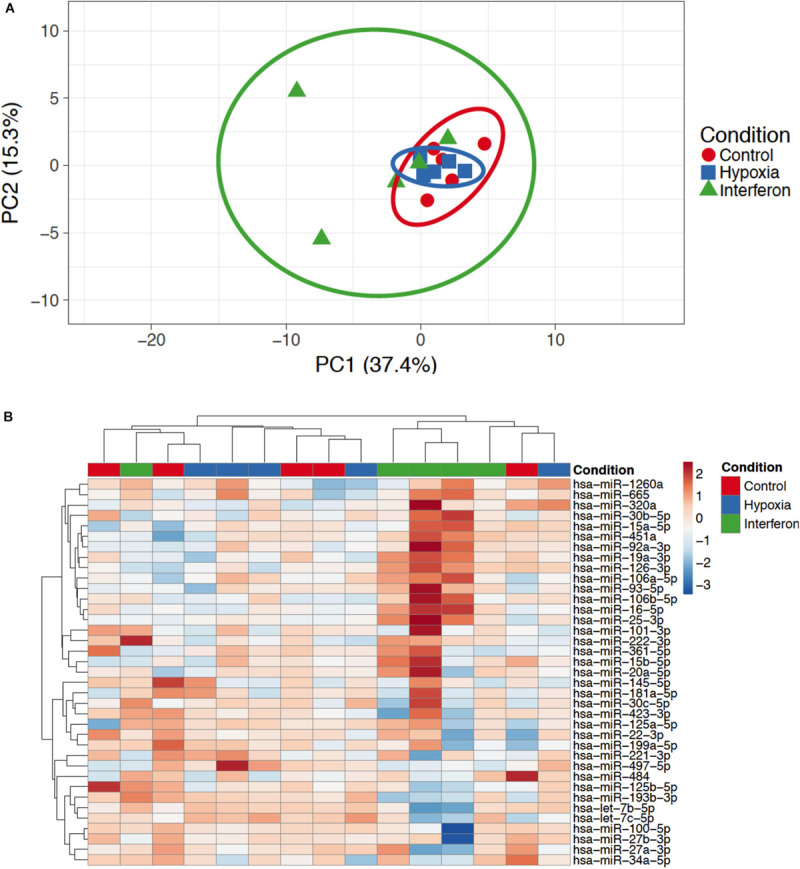
**(A)** Principal component analysis. Original values were ln(x + 1)-transformed. Unit variance scaling was applied to rows; SVD with imputation was used to calculate principal components. *X* and *Y* axes show principal component 1 and principal component 2 that explain 37.4 and 15.3% of the total variance, respectively. Prediction ellipses are such that with probability 0.95, a new observation from the same group will fall inside the ellipse. *N* = 15 data points. **(B)** Heatmap showing the expression of the miRNA in extracellular vesicles in Control Hypoxia and Interferon groups. Original values were ln(x + 1)-transformed. Rows were centered; unit variance scaling was applied to rows. Both rows and columns were clustered using correlation distance and average linkage.

Eighty-one miRNAs were detected in at least one of the pools (Control, IFN, or HYP); only 46 of them were detected in each sample. Forty-one miRNAs were quantified since five reference miRNAs were necessary to reach Genorm requirements for the normalization (hsa-let-7a-5p, hsa-miR-21-5p, hsa-miR-23a-3p, hsa-miR-23b-3p, and hsa-miR-24-3p). Three miRNAs had Ct values that did not allow reliable quantification. The PCA performed on the 38 remaining miRNA could not separate the Control, IFN, and HYP groups (Figure 2A); this was further confirmed by the poor clustering found on the heatmap (Figure 2B).

### Individual miRNA Expression in EV Is Weakly Affected by Priming

**FIGURE 3:**
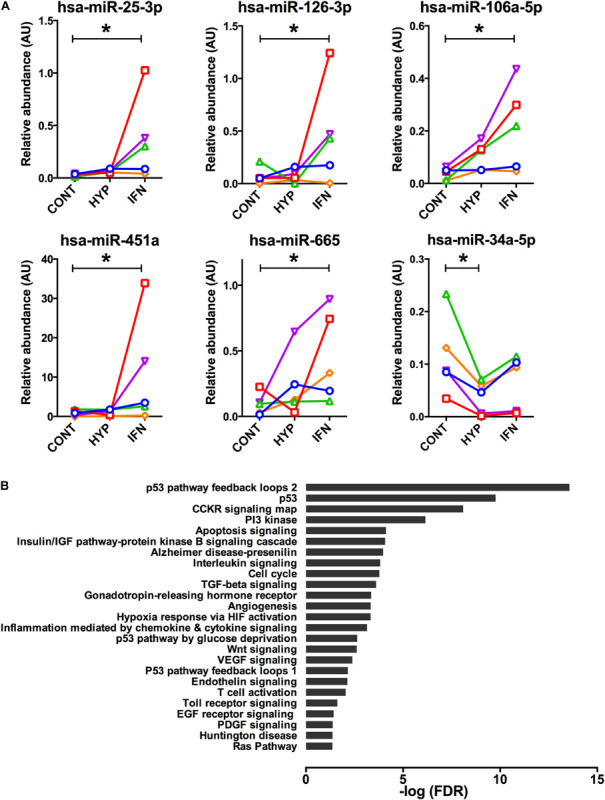
**(A)** Significantly altered miRNA levels in extracellular vesicles of Control, Hypoxia, and Interferon-treated MSC are shown. miRNAs were measured by RT-qPCR and results were normalized with five internal reference miRNA. Friedmann ANOVA was used to assess a global effect (not shown), when significant Dunn’s multiple comparison test was used for inter-group comparison *p < 0.05. **(B)** The target genes of the five miRNAs increased in response to IFN were identified with MirtarBase 8.0 and an enrichment analysis in biological pathways was performed using Panther classification system 14.0.

The individual analysis of the 38 miRNA showed that a limited number of miRNA were affected by the priming. Five miRNA (hsa-miR-25-3p, hsa-miR-106a-5p, hsa-miR-126-3p, hsa-miR-451a, and hsa-miR-665) were significantly upregulated in response to interferon preconditioning (Figure 3A). The target genes of these three miRNAs were determined and an enrichment analysis was performed. Biological pathways relevant to tissue repair and immunomodulation were identified (Figure 3B).

Only hsa-miR-34a-5p was significantly reduced by HYP (Figure 3A).

**FIGURE 4:**
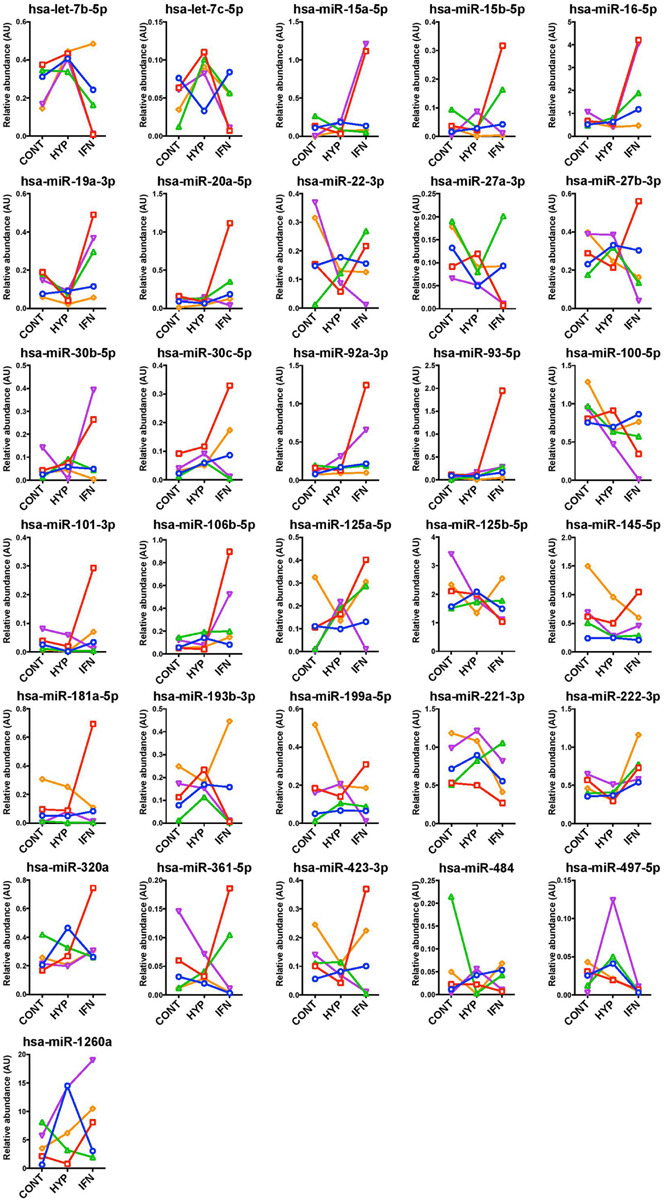
Unaltered miRNA levels in extracellular vesicles of Control, Hypoxia, and Interferon-treated MSC. miRNAs were measured by RT-qPCR and results were normalized with five internal reference miRNAs.

Most miRNA levels in MSC-EV remained unchanged in response to interferon or HYP priming (Figure 4).

## Discussion

Several studies have proposed that MSCs-secreted EV can improve tissue repair by delivering their molecular cargo to the injured tissue. EV contain various molecules including proteins and RNA. Because miRNAs modulate the expression of up to 60% of human genes (Lewis et al., 2005; Friedman et al., 2008), their specific role in the therapeutic effects of EV treatment has been explored. Individual miRNAs have been shown to play a central role in brain or myocardium repair after an ischemic injury (Xin et al., 2013; Luther et al., 2018). However, it has been suggested that miRNA could act, not only as individual molecules, but also collectively; therefore, the miRNA landscape of BM-MSC-EV has been examined (Ferguson et al., 2018). More than 150 miRNA have been identified in MSC-EV; however, 23 of them accounted for 80% of the total amount. The bioinformatics analysis of the predicted targets of these 23 miRNA showed that they were involved in biological pathways relevant to tissue repair such as angiogenesis, TGFβ, and PDGF signaling. In the work by Fergusson, the EV were isolated by precipitation whereas we isolated EV by ultracentrifugation. Although this may introduce some differences in EV populations and/or co-purified molecules, our qPCR profiling detected 28 miRNAs common to the 50 most abundant described and eight common to their 10 most abundant (hsa-miR-23a-3p, 451a, 125b-5p, 199a-3p, 100-5p, 21-5p, let-7a-5p, and let-7b-5p). Among the most expressed miRNA reported by Fergusson, only hsa-miR-144-3p and -630 were negative in our profiling. This suggests that BM-MSC EV miRNA landscape is specific and robust. Yet, it should be mentioned that neither the nanostring technology used by Fergusson nor our qPCR panels allow a comprehensive identification of the miRNA since they are based on probes or primers. Therefore, more miRNAs could be identified in RNA-Seq studies. Furthermore, the miRNA expression within MSC EV could vary according to the tissue and/or species, as suggested by the very different profile described, for example, in porcine adipose-derived MSC (Meng et al., 2018). There are currently several methods used to isolate EV from conditioned media; although ultracentrifugation is widely used in the literature, some limitations should be mentioned. The most important one is that UC could co-purify large protein aggregates that may bind free miRNA in a non-specific manner. This has been reported to induce variations in the miRNA profile from serum EV (Andreu et al., 2016; Buschmann et al., 2018). Although this may have an impact on our results, similar limitations are described for precipitation and filtration methods.

### Effect of Priming

Because of their high plasticity *in vitro*, priming of MSC during the culture phase has been proposed to improve their biologic activity or provide them new functions, without altering their safety profile (Guess et al., 2017). Numerous primings have been studied, including inflammatory (TNFα, IFNγ, IL1β) or hypoxic conditions (Andreeva et al., 2017). IFNγ preconditioning has been widely studied and improved MSC efficacy in models where an immunomodulatory effect of MSC is expected, such as graft versus host disease (Polchert et al., 2008; Tobin et al., 2013), colitis (Duijvestein et al., 2011), or sepsis (Baudry et al., 2019). A well-documented effect of both inflammatory and hypoxic MSC priming is the modulation of their secretome, with altered cytokines, chemokines, enzymes, or growth factors secretion (for review, see Ferreira et al., 2018). An important part of those molecules can be found as soluble factors or within EV. Less is known about the capacity of priming to alter EV content, yet some studies suggest that inflammatory preconditioning (IFNγ) increases IL-10 and IDO in umbilical cord-derived MSC EV (Zhang et al., 2018), showing that priming could help engineering specific MSC EV. Because miRNAs are thought to be important actors of MSC EV in cell therapy, we hypothesized that priming could significantly modulate miRNA content. We studied two well-documented priming, IFNγ and HYP, and found only limited effects of these conditions on highly expressed miRNA. The PCA and the clustering of the miRNA results showed that the priming used here induced no major alteration of the miRNA landscape and only minimal differences could be found between primed and naive groups.

Yet, taken individually, five miRNAs were significantly upregulated in IFN-primed MSC EV and one miRNA was downregulated in HYP MSC EV. The bioinformatics analysis first consisted in identifying the experimentally validated targets of selected miRNAs in miRBase and then analyzing the targets in a gene enrichment database (Panther Classification System). This approach was chosen because specific miRNA enrichment databases are very much annotated in the field of cancer, which was not relevant to our study. We identified 106 target genes; many were involved in pathways relevant to tissue repair including “p53,” “cell cycle,” “TGF-beta signaling,” “angiogenesis,” or “PDGF signaling.” Some pathways were also relevant to inflammation and immunomodulation such as “interleukin signaling,” “inflammation mediated by chemokine and cytokine signaling,” or “Toll receptor signaling.” However, because of the limited number of miRNA affected in our conditions, it is unclear whether priming could have a significant impact on MSC EV functionality mediated by miRNA. Based on sequencing of endometrial MSC-derived EV, Marinaro et al. found that interferon priming could significantly alter only four miRNAs, but the PCR analysis did not confirm this result. No differences were found in the five miRNAs we identified, possibly due do a different cell type but also to the priming itself (interferon 3 ng/ml for 6 days). However, congruent with our data, the effect of priming was found to be far less important on EV miRNA that on protein content (Marinaro et al., 2019). No specific effect of the five miRNAs modulated in our MSC EV could be found in the literature, therefore we could not identify and perform any specific *in vitro* functional tests that could be representative of their activity. This may be a limitation in this study, but we think that because of the small number of miRNA modulated by priming and because other EV molecules also play important roles, such tests would have been of limited interest.

### Inter-Individual Variability

In our study, we used five different donors and found that the MSC EV miRNA content response to priming substantially differed between donors. Only two out of five donors displayed very different miRNA signature in EV in response to IFN priming, suggesting they were highly responsive to this specific cytokine. These two donors separate from other individual in the PCA in the IFN group (Figure 2A). Taken individually, several miRNAs were strongly affected by the priming only in some donors suggesting a high variability in individual response to IFN. Only five miRNAs were consistently increased in all donors in response to IFN, thereby reaching statistical significance. Similarly, only one miRNA was consistently decreased in response to HYP. Important differences in MSC functionality have been described according to their tissue source. Yet, within cells originating from the same tissue, for example, bone marrow, differences still exist that mostly depend on the donor. Growth properties, differentiation potential, or secreted factors are not identical between individuals; they can sometimes be explained by age, gender, or MSC stemness, and may explain some inconsistencies found in the literature (Phinney et al., 1999; Siddappa et al., 2007; Siegel et al., 2013). Priming is sometimes considered an interesting tool to overcome the inter-donor variability in MSC functionality thereby improving their clinical use (Noronha et al., 2019). Yet not all donors respond similarly to inflammatory priming suggesting that cell preconditioning needs to be tested and optimized for each individual indication, and that donors may have to be carefully selected when an allogeneic treatment is considered (François et al., 2012; Jin et al., 2016). Changes in secreted factors such as growth factors chemokines and cytokines have been widely studied in MSC secretome. Our findings show that miRNA seem to behave the same as other factors, with some variations in the expression in response to priming stimuli, in a donor-dependent manner. The mechanisms underlying these differences are not known. In a study examining adipose tissue MSC-derived EV, a lower inter-individual variability in the miRNA response to IFN was reported, suggesting that the tissue of origin may contribute to the response observed (Ragni et al., 2020).

## Conclusion

There is an increasing interest in using MSC EV as a surrogate for MSC therapy in various applications such as immunomodulation and tissue repair. We show here that in human bone marrow-derived MSC, two classical priming conditions have only a limited effect on EV miRNA landscape, suggesting that the beneficial effects of such preconditioning on cell biological activity do not rely on EV miRNA. Yet, because an important inter-individual variability was found, more research is needed to understand how cell priming could help improve MSC EV functionality mediated by miRNA.

## Data Availability Statement

The raw data supporting the conclusions of this article are available in a public database (GEO database, #GSE156919).

## Ethics Statement

Ethical review and approval was not required for the study on human participants in accordance with the local legislation and institutional requirements. The patients/participants provided their written informed consent to participate in this study.

## Author Contributions

JP, J-JL, and SB conceived and designed the experiments. KL, JP, M-EG, MG, and PM performed the experiments. JP, KL, PM, and SB analyzed the data. JP and SB wrote the manuscript. All authors reviewed and edited the manuscript.

## Conflict of Interest

The authors declare that the research was conducted in the absence of any commercial or financial relationships that could be construed as a potential conflict of interest.

## Footnotes

1 Funrich.org
